# Crystallisation in a two-dimensional granular system at constant temperature

**DOI:** 10.1038/s41598-021-96099-9

**Published:** 2021-08-16

**Authors:** M. Ledesma-Motolinía, J. L. Carrillo-Estrada, F. Donado

**Affiliations:** 1 Instituto de Física “Luis Rivera Terrazas”, Benemérita Universidad Autónoma de Puebla, Puebla, 72570 Mexico; 2 Instituto de Ciencias Básicas e Ingeniería, Universidad Autónoma del Estado de Hidalgo-AAMF, Pachuca, 42184 México

**Keywords:** Structure of solids and liquids, Phase transitions and critical phenomena

## Abstract

We study the crystallisation processes occurring in a nonvibrating two-dimensional magnetic granular system at various fixed values of the effective temperature. In this system, the energy loss due to dissipative effects is compensated by the continuous energy input coming into the system from a sinusoidal magnetic field. When this balance leads to high values of the effective temperature, no aggregates are formed, because particles’ kinetic energy prevents them from aggregating. For lower effective temperatures, formation of small aggregates is observed. The smaller the values of the applied field’s amplitude, the larger the number of these disordered aggregates. One also observes that when clusters form at a given effective temperature, the average effective diffusion coefficient decreases as time increases. For medium values of the effective temperature, formation of small crystals is observed. We find that the sixth bond-orientational order parameter and the number of bonds, when considering more than two, are very sensitive for exhibiting the order in the system, even when crystals are still very small.

## Introduction

For a long time, crystallisation has attracted scientific attention, particularly through attempts to understand the nucleation mechanisms. Crystallisation under certain conditions can be seen as a phase transition from a disordered structure to a periodically ordered one. This process can be observed in some systems while cooling down, going from a fluid phase to a solid one. It is also observed in systems going from disordered solid states to ordered states when they are subjected to annealing. Crystallisation begins with the formation of a small core, a nucleus. The classical homogeneous nucleation theory (CNT)^1,2^ states that due to particle concentration fluctuations, small aggregates are formed. If these are smaller than a certain critical size, they disintegrate, but if they are bigger than the critical size, they become stable and grow. According to CNT, once an aggregate becomes stable, it grows by further aggregation, and its crystalline structure will determine the structure of the final aggregate. The gradual growth of the structure is due to the incorporation of new particles in minimal energy positions of the lattice. Crystallisation processes can also start with heterogeneous nucleation, whereby the nucleus is formed on impurities or on the borders of the system. Heterogeneous nucleation occurs more frequently than homogeneous nucleation, and it has been observed in confined systems^3,4^ and in cases where the walls that limit the system are modified to induce nucleation^5^.

It is well known that better understanding of crystallisation will facilitate the control of a wide variety of phenomena. At the atomic scale, crystallisation processes are studied by indirect methods due to their microscopic nature. Furthering our in-depth understanding of crystallisation processes requires direct studies capable of describing them at the level of single particles. These kinds of studies would allow us to validate nucleation theories; in particular, they could provide evidence to support or discard non-classical nucleation theories. No studies provide sufficient spatial and temporal resolution to describe crystallisation processes at the molecular level, although some notable progress in that direction has been achieved^6–9^. For many years, colloids have served as models to describe the crystallisation process in two^10^ and three dimensions^11–14^. For instance, it has been found that under certain conditions, non-classical two-step nucleation occurs^15,16^. This result is contrary to what is proposed by CNT, where the crystallisation process occurs in a single step. A two-step mechanism non-classical theory of nucleation establishes that a cluster of particles of sufficiently large size may arise with a disordered structure, which subsequently rearranges and gives rise to a crystalline nucleus^17,18^.

Even though colloidal systems provide some insight into the true nature of crystallisation, they present some limitations. For instance, particle concentration and effective temperature are coupled: the effective temperature is proportional to the inverse of the particle concentration. It is desirable to control particle concentration and effective temperature independently. To overcome some of the issues arising when modelling crystallisation with colloidal systems, and to improve our knowledge, we have been using granular matter systems, in which particles are clearly athermal, because they are not affected by thermal fluctuations^19^. To introduce something analogous to an effective temperature in these systems, it is necessary to inject energy through some mechanisms.

Most experiments involving granular systems use mechanical vibration to fluidise the set of particles, thus controlling the effective temperature. In some other cases, periodic shearing has been used to induce structural changes in the system^20–22^. Despite being dissipative systems, granular models present structural configurations equivalent to systems in equilibrium^23,24^. Colloidal systems and granular systems allow us to follow the individual trajectories of the constituent particles and therefore to obtain the structural and dynamic properties. In Ref.^21^, a combination of vibration and shearing was used to produce reversible phase transitions between crystalline phases. In Refs.^23,25^, a vibrating granular system was used to measure the structural changes during the crystallisation and glass transition processes in a two-dimensional system.

Previously, we have used a nonvibrating granular system under a sinusoidal magnetic field as a model to investigate, at a particle level, some pattern formation processes in fluids. According to the theory of Ornstein–Uhlenbeck processes, the resulting motion has all the Brownian motion characteristics, as we have shown in Refs.^24,26^. We have studied the behaviour of the system when it suffers a sudden quenching. In those experiments, particles were under a sinusoidal magnetic field with an offset which produces effective repulsive interaction between particles. This effective repulsion prevents particles from aggregating, and then the system is able to model a glass-transition-like transformation. We have also studied crystallisation by using a lens^27^, and a tilted planar cell^28^. In those experiments, we have produced a cooling down process by decreasing the magnetic field using a step-down-like signal.

A special characteristic of the granular nonvibrating system is that particle concentration and effective temperature can be independently controlled for low and medium particle concentrations, which is a very attractive advantage over other macroscopic systems. We can carry out experiments at a fixed particle concentration while varying the effective temperature. It has been shown that at low particle concentrations, the effective temperature is directly proportional to the amplitude of the applied magnetic field^24^. By adding a constant magnetic field, the intensity of the interactions between particles can be controlled. When the system is under a sinusoidal magnetic field with an offset, the effective repulsion prevents particles from aggregating. Without the offset and below a magnetic threshold, particles tend to aggregate. As the particle concentration increases, the size of the aggregates grows. Depending on the effective temperature, the aggregates are more or less ordered. If the temperature is relatively high, these aggregates disintegrate, but below a certain threshold, the aggregates are stable and tend to grow.

In the present work, we analyse aggregates formed under a long-term regime at a constant temperature; we considered several effective temperature values. Because the aggregates are formed by a few particles, we determine the structural characteristics by using the sixth bond-orientational order parameter and the number of bonds. To standardise, we start the experiments at a high temperature and then abruptly set the desired temperature in the system. The experiments are recorded during a relatively long period to observe whether aggregates are formed or not, and to determine the structural characteristics of the clusters if aggregation occurs.

## Results

The main advantage of using this granular system to study crystallisation processes is that simple optical microscopy allows observation, in real time, of complex phenomena in which energetic and entropic interactions generate the structure patterns. For instance, one may observe at fixed particle concentrations the crystallisation process for several fixed effective temperature values. Under these conditions, we observe that between high temperatures, at which the kinetic energy of particles prevents stable aggregates forming, and low temperatures, at which numerous disordered aggregates occur, medium temperatures promote the formation of aggregates with a more ordered structure. It is observed that most of the aggregates are small crystals.

**Figure 1:**
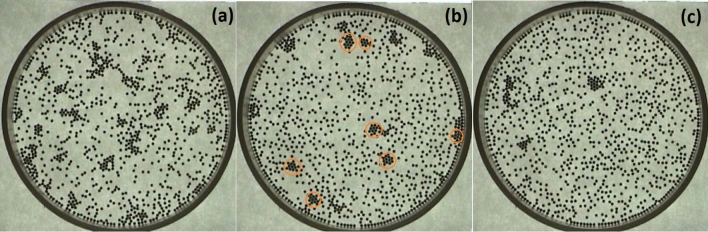
Final configurations at different amplitudes of the applied magnetic field. From (**a**) to (**c**), the amplitudes are 24.87 G, 33.32 G and 44.09 G (from series 3). The hexagonal close packed structures that are locally formed at 33.32 G are enclosed in orange circles.

We carried out three series of experiments (series 1, series 2 and series 3) for each value of the effective temperature. The configurations of the granular system after 30 mins at three different temperatures (from series 3) are shown in Fig. 1. The circular walls of the cell prevent particles from escaping. It is observed that a ring of particles is formed at the wall container. Particles near the circular wall suffer more collisions from particles inside the sample than those near the wall. Therefore, the entropic interactions are the origin of the formation of the ring^29–32^. No aggregates are formed at a relatively high effective temperature, but below an effective threshold temperature, they start to form. The number of aggregates increases as the effective temperature decreases. It is observed that the lower the effective temperature, the bigger the disorder of the inner cluster structure. Some aggregates are anchored to the cell border, when they are crystalline this is due to heterogeneous nucleation^33–36^. This effect increases as the temperatures are increased. For lower temperatures, the formation of aggregates separated from the wall is observed, when the aggregates are ordered this is preceded by a two-dimensional homogeneous nucleation^37–39^.

**Figure 2:**
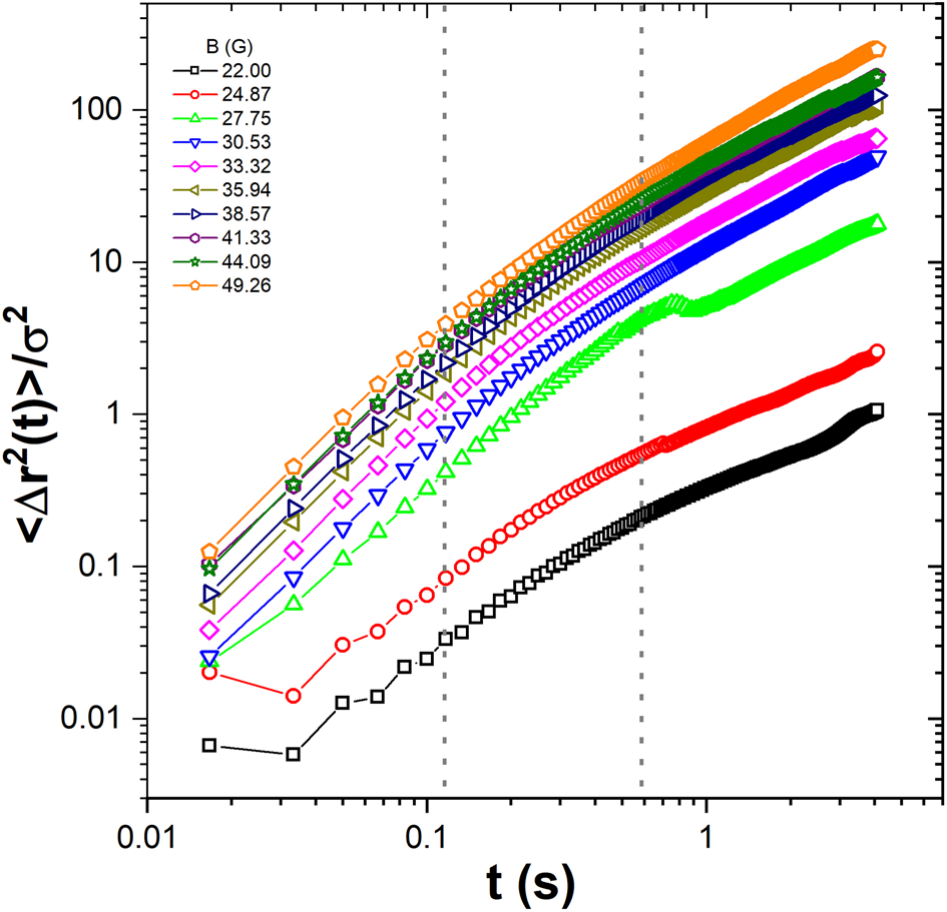
The mean square displacement as a function of time for several magnetic fields for the first set of experiments (series 1). The vertical dotted lines indicate the time window where the linear fit is done.

**Figure 3:**
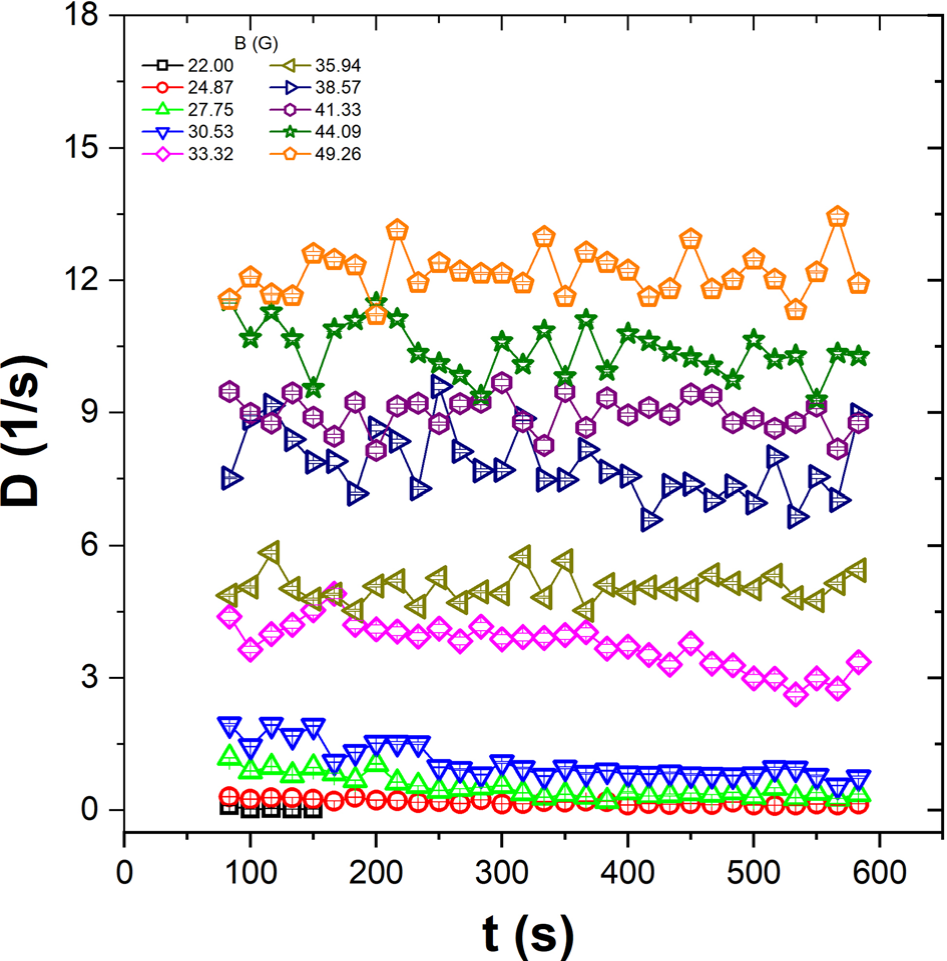
The time evolution of the diffusion coefficient for different amplitudes of the magnetic fields, for the first set of experiments (series 1).

We characterised the dynamical nature of the system by using mean square displacement, $<\Delta r^{2}\hbox {(t)}>=<|r(t)-r(0)|^2>$, $\Delta r$ is measured in particle diameter units $\sigma$, for several values of the applied magnetic field. The results are shown in Fig. 2. The first six points correspond to the quasi-ballistic regime^40^, and these points are not used to calculate the diffusion coefficient, which was determined by using the following 30 points. In the range of the effective temperature here explored, the $<\Delta r^{2}\hbox {(t)}>$ curves show that particles’ behaviour is diffusive, except for the first six points. Moreover, as the effective temperature increases, the slope of the curve also increases. This means that when the effective temperature increases, the particles move at a higher speed. To show this, we determined the diffusion coefficient. The effective diffusion coefficient (*D*) was obtained by means of a linear fit of the $<\Delta r^{2}\hbox {(t)}>$ (ignoring the first points) and from the relation $<\Delta r^{2}\hbox {(t)}> \sim 4D t$. The time interval in which the effective diffusion coefficient is calculated is shown in Fig. 2, delimited by vertical dotted lines. Figure 3 shows the time evolution of the diffusion coefficient over the range of magnetic fields (effective temperature) analysed for one of the sets of experiments (series 1). At high temperatures, the diffusion coefficient remains almost constant, except for some fluctuations. At lower temperatures, it decreases as a function of time. For low and medium temperatures, we observe that after 200 s, *D* decreases slightly and then remains constant until the final measurement time.

**Figure 4:**
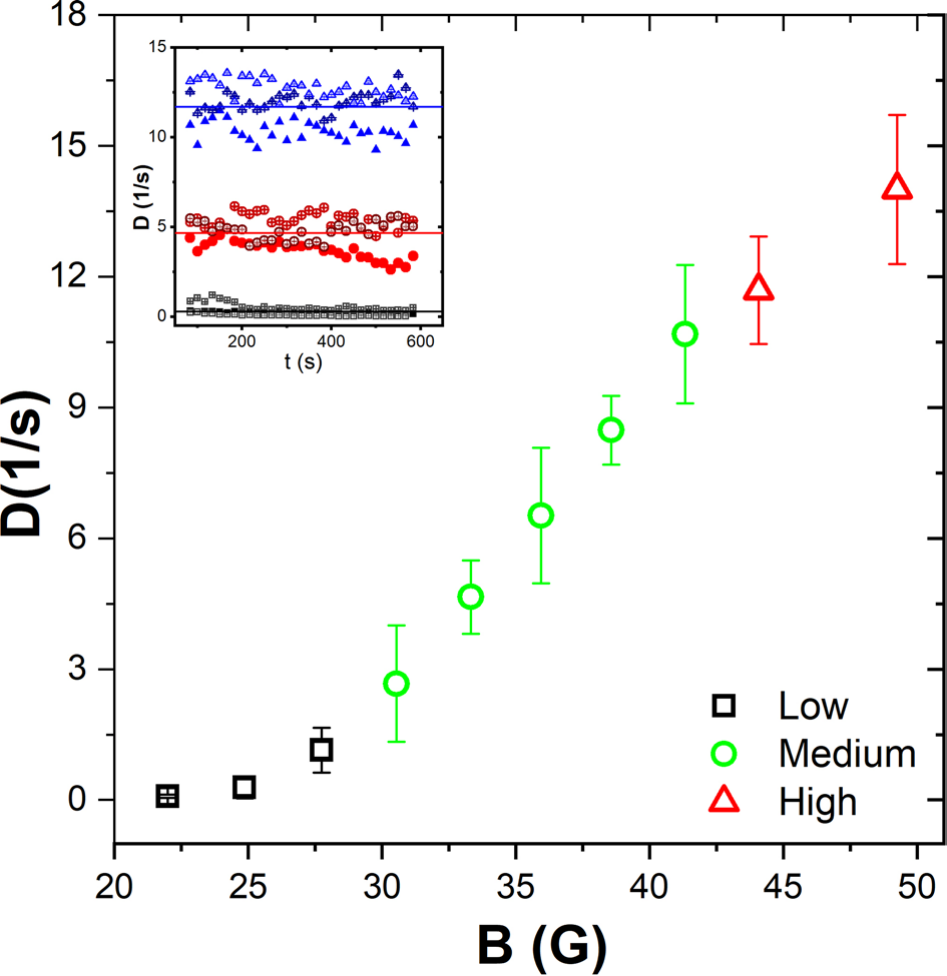
The average values of the effective diffusion coefficient as a function of the magnetic field. The symbols in black represent the low-temperature regime. Green symbols are for the medium temperatures. Finally, red symbols are for high-temperatures. The inset illustrates the similarity between the temporal evolution of the effective diffusion coefficient at 24.87, 33.32, and 44.09 G. The horizontal lines indicate the average value.

Figure 4 shows the behaviour of the average diffusion coefficient as a function of the amplitude of the applied field—namely, our effective temperature. At least three regions in the behaviour of the average *D* are observed, clearly distinguished by their slope at high, medium and low temperatures. At low temperatures (black symbols), the particles move more slowly and form aggregates all over the surface, mostly disordered. In the medium region (green symbols), the particles move at a higher speed, with the formed aggregates being more organised and tending to form compact hexagonal arrangements, which are mostly located in the central region of the cell. Finally, in the high-temperature region (red symbols), the particles move quickly, and their kinetic energy almost prevents cluster formation, which is observed only in the formation of very few small ordered aggregates. At even higher temperatures, the only observed aggregates are anchored to the wall of the cell, forming part of a ring-like structure. The inset in Fig. 4 shows the raw data, and its arithmetic averaged for the three values of the effective temperature.

We have found that for granular systems such as ours, to evaluate the degree of order of the aggregates as a function of the number of neighbours (*n*) around a given particle, the sixth bond-orientational order parameter $\Psi _{6}$ works well^27,41^. Details are discussed in the “Methods” section.

**Figure 5:**
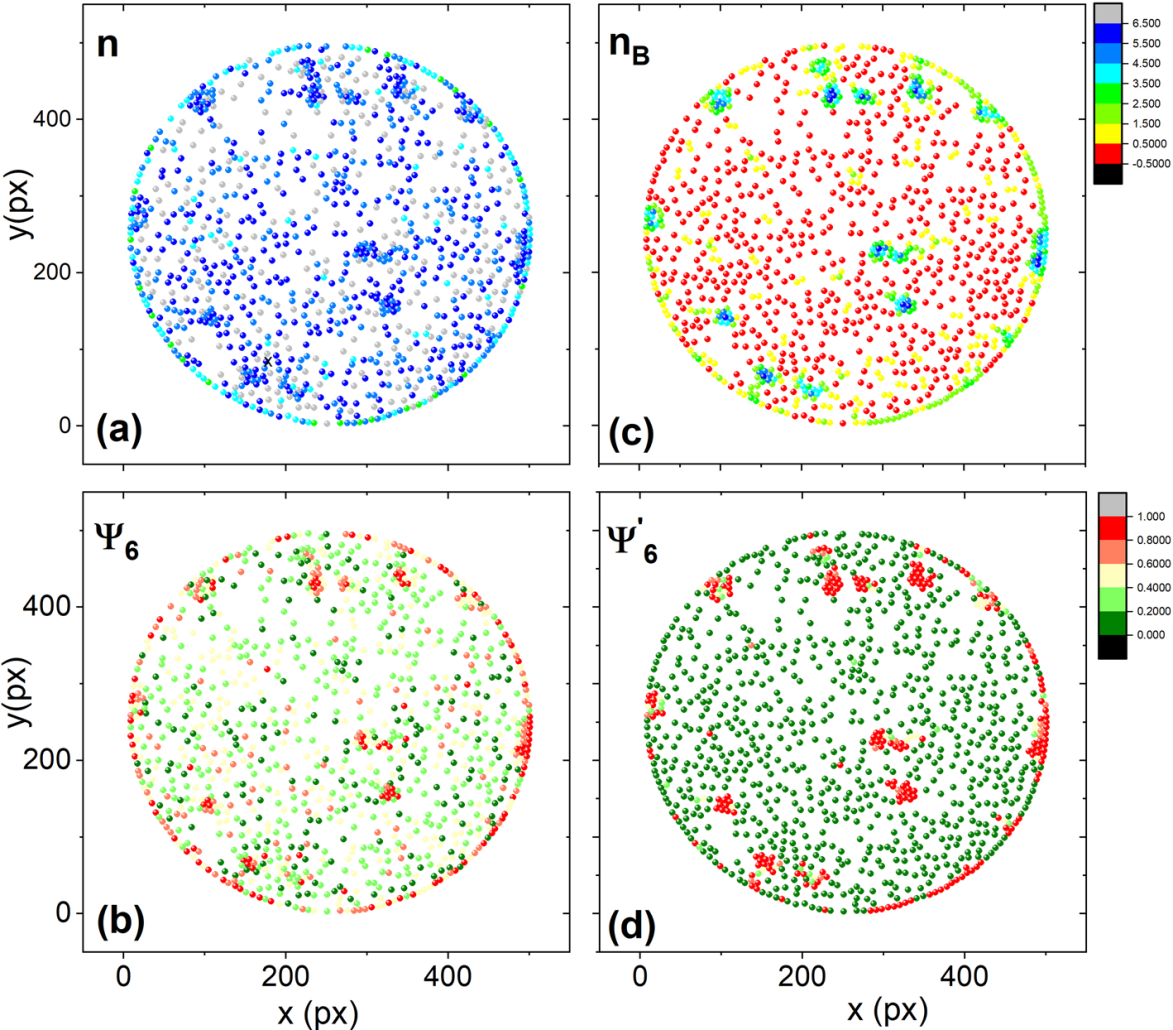
It is shown the final configuration of particles, (**a**) points are coloured according to the number of their neighbours n, and (**b**) particles are coloured according to the sixth orientational order parameter $\Psi _6$. (**c**) points are coloured according to the number of bounded neighbours n_B_. (**d**) points are coloured according to the sixth bond orientational order parameter $\Psi ^{'}_{6}$. The experiment corresponds to the effective temperature of 33.32 G in series 3.

To discuss the order and compactness of the aggregates’ structure, we choose an effective temperature in the medium range, where aggregates are more ordered, and most of them are crystalline. In Fig. 1b, it can be seen several ordered aggregates, both at the centre and at the cell’s border. In Fig. 5a, particles are coloured according to the number of neighbours; the dark blue colour represents those particles with six neighbours. Comparing the picture and Fig. 1b, we observed that many particles have six neighbours even though some of them are far away from the particle. This is a characteristic of determining neighbours using the Delaunay triangulation or its dual graph, the Voronoi diagram^42^. Based on these neighbours, the sixth bond-orientational order parameter $\Psi _6$ is evaluated. Figure 5b shows particles coloured according to this quantity. It is observed that some particles are indicated as disordered even though they belong to an ordered cluster. This is a direct consequence of taking into account all the neighbours obtained through the Delaunay triangulation. We observed that these parameters are not sensitive to small ordered aggregates. For characterisation of those small ordered aggregates that form a compact hexagonal network, the bounded neighbours ($n_B$) are calculated—i.e., those particles in contact with a particle within a distance of one particle diameter $r=\sigma$. Based on these bounded neighbours, a new parameter is introduced ($\Psi ^{'}_{6}$); this is similar to $\Psi _{6}$ but measured considering only bounded neighbours. The perfect sixth bond-orientational order configuration is reached when angles between the nearest neighbours are multiples of $\pi /3$. Considering the above configuration, we plot the number of the bounded neighbours, $n_B$, and the parameter, $\Psi ^{'}_{6}$, in Fig. 5c and d, respectively. Those particles having six neighbours in contact are coloured dark blue, while particles without neighbours in contact are red coloured. Also, we found that the parameter $\Psi ^{'}_{6}$ adequately recognises the ordered aggregates. These clusters appear enclosed in orange circles in Fig. 1b.

**Figure 6:**
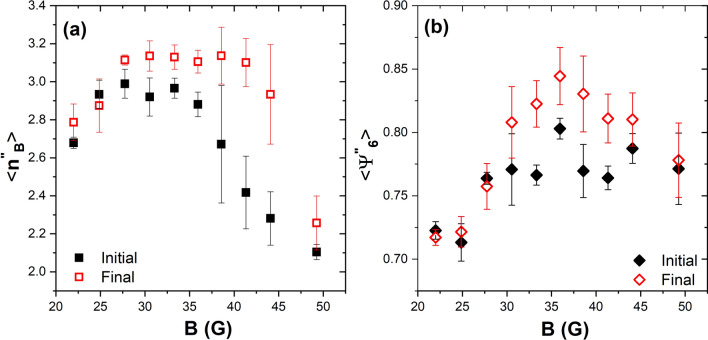
(**a**) The average number of the bounded neighbours, $<n^{''}_{B}>$, considering two or more particles in contact with a given particle for the initial and the final configurations as a function of the effective temperature. (**b**) The behaviour of the average values of the sixth bond orientational order parameter, $<\Psi ^{''}_{6}>$, for two o more bounded neighbours for each particle as a function of the magnetic field. The parameter is depicted for the initial and the final configurations.

When the number of bounds is just one, a particle could be unstable and is likely to abandon the aggregate. That is why we further modified the sixth bond-orientational order parameter by considering only those particles whose number of bounded neighbours is equal to or greater than two, represented by the parameter $n^{''}_{B}$. The arithmetic average of the three values of $n^{''}_{B}$ for the three series, at different magnetic field values, is shown in Fig. 6a. We compare the initial configuration (measured at short time intervals) to the final configuration (measured at long time intervals). This figure shows behaviour similar to the effective diffusion coefficient as a function of effective temperature. At low temperatures, the number of particles with two or more neighbours in contact is small; as the temperature increases, the number of the bounded neighbours in contact increases, which allows the formation of compact hexagonal networks that subsequently lead to bigger ordered aggregates. Finally, at high temperatures, the particles move with a higher velocity and form a few aggregates; however, these are made up of hexagonal networks. The corresponding sixth bond-orientational order parameter, considering two or more bounded neighbours, is denoted $\Psi ^{''}_{6}$. This parameter allows the identification of the most stable and well-ordered formations. Figure 6b shows the average of $\Psi ^{''}_{6}$ as a function of the magnetic field. From this figure, it is more evident that there are three regimes in the behaviour of the structure of the aggregates. Thus, it supports the observation that at medium temperatures, the aggregates are well ordered, forming hexagonal lattices.

**Figure 7:**
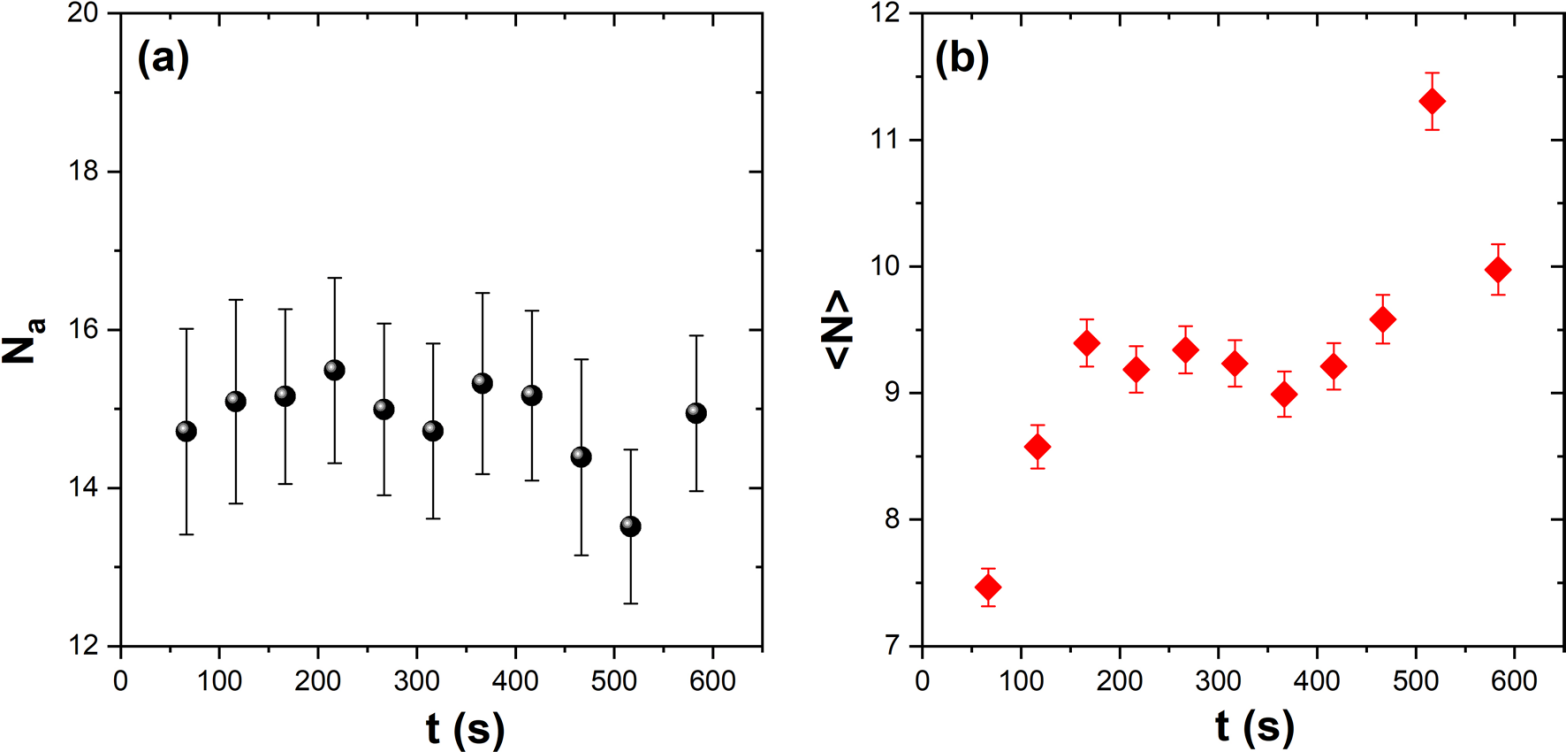
(**a**) Time evolution of the average number aggregates $N_a$, and (**b**) temporal evolution of the aggregates average size $<N>$ at the medium-effective temperature produced by the field amplitude 35.94 G in series 3.

**Figure 8:**
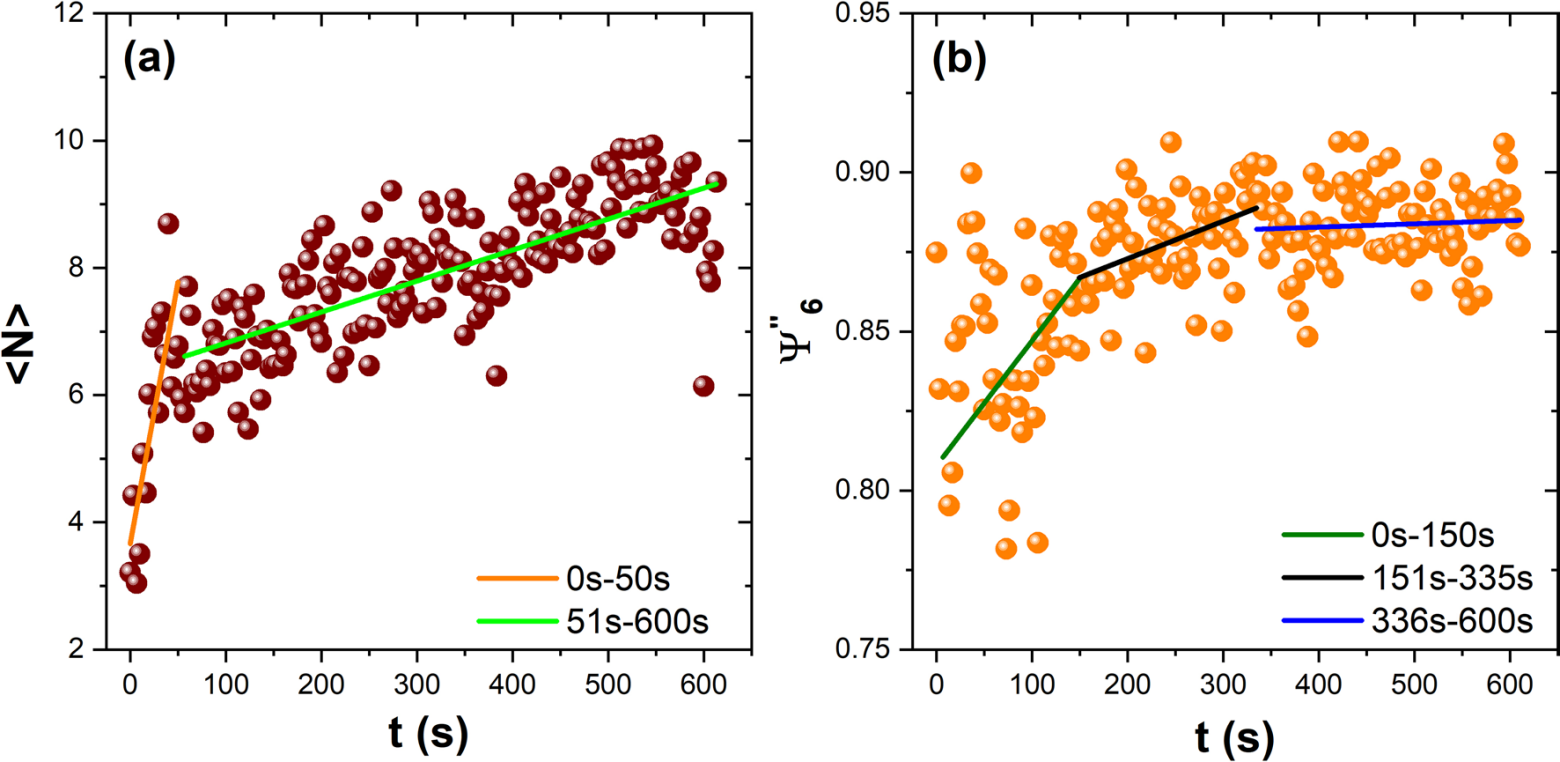
(**a**) Temporal evolution of the aggregates average size, and (**b**) the sixth bond orientational order parameter $\Psi ^{''}_{6}$ as a function of time at the medium-effective temperature produced by the field amplitude 35.94 G in series 3. The solid line corresponds to a linear fit to represent the ratio of increments.

As mentioned, the aggregates are stable and only increase in size as a function of time. To demonstrate this fact, an intermediate temperature is considered, and the time evolution of the average number of aggregates, $N_a$, and the average size of aggregates, $<N>$, were analysed. Figure 7a shows that the number of aggregates remains constant over the measurement interval, which means that the aggregates that overpass the critical size will never disaggregate. On the other hand, Figure 7b shows the temporal behaviour of $<N>$. The largest observed clusters are about *N* = 30, where *N* is the particle number. We notice that the aggregates at the early stage grow quickly and then hold their average size, before finally growing again. Experimentally, we observe that the disordered aggregates diminish in size while the ordered ones increase, in such a way that the average size remains constant as it leads the plateau of the curve. The above is confirmed when we only analyse the time evolution of the ordered aggregates ($\Psi ^{''}_{6}$ > 0.8). Figure 8a shows the behaviour of $<N>$ with a high degree of order as a function of time. Two characteristic growth ratios are found. In the first one (0–50 s), the aggregates form rapidly and reach the critical size; in the second one, they exhibit a lower rate of growth. Figure 8b shows the time evolution of $\Psi ^{''}_{6}$. The aggregates experience a fast ordering until 150 s, and then they rearrange, reaching a higher degree of order. After that (335 s), the aggregates only grow epitaxially.

**Figure 9:**
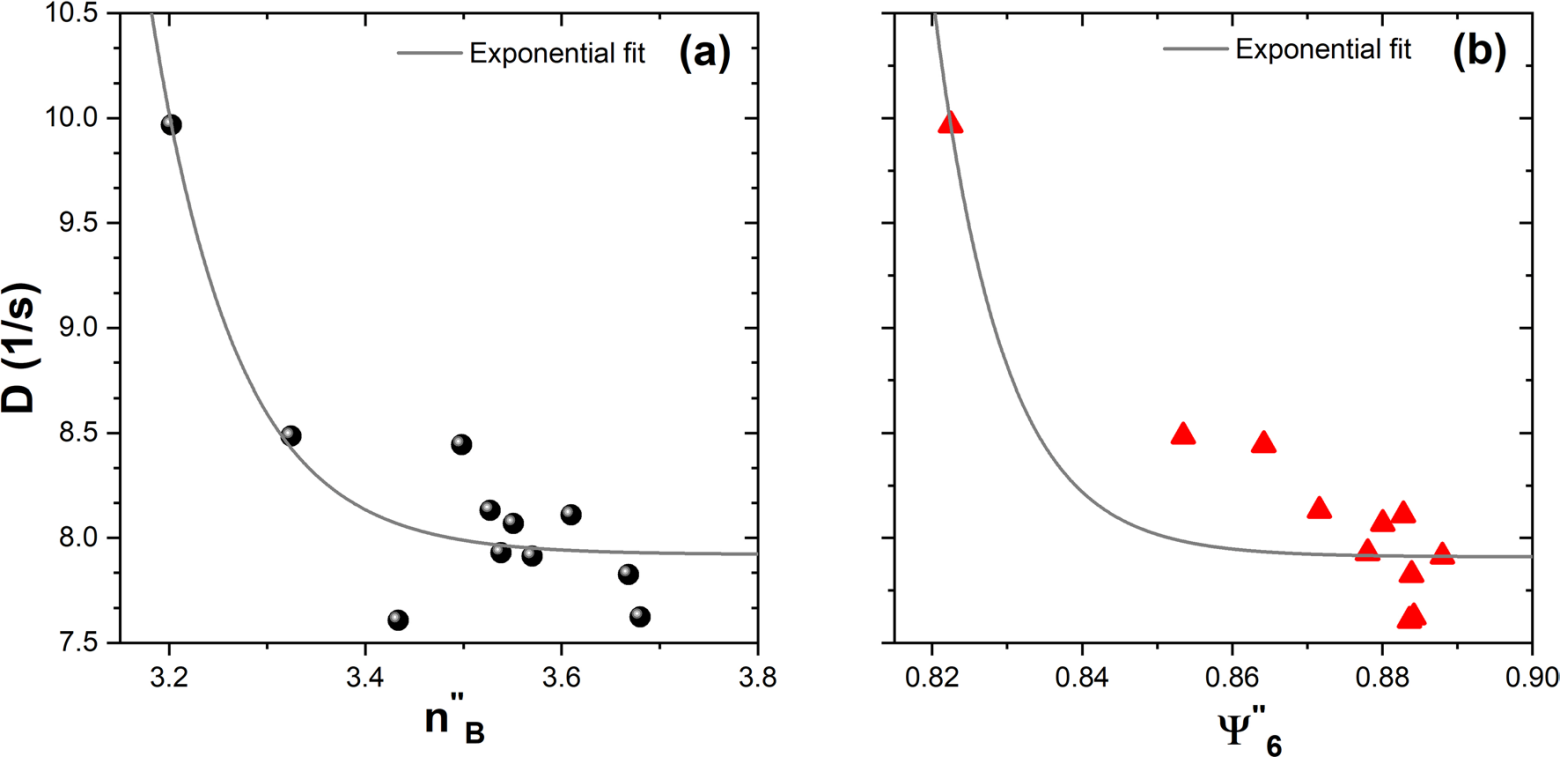
(**a**) The effective diffusion coefficient as a function of the number of the bounded neighbours $n^{''}_{B}$, and (**b**) the effective diffusion coefficient as a function of the sixth bond orientational order parameter $\Psi ^{''}_{6}$ at the medium-effective temperature produced by the field amplitude 35.94 G in series 3. The solid line corresponds to an exponential fit only to indicate the behaviour of the diffusion coefficient.

This idea that ordered aggregates only grow in size by the adhesion of free particles is consistent with the effective diffusion coefficient decreasing when the aggregates’ size increases. To demonstrate this statement, Fig. 9a and b show the relationship between the effective diffusion coefficient *D* and the bond-orientational order parameters that characterise the degree of order of the aggregates, $n^{''}_{B}$ and $\Psi ^{''}_{6}$, respectively. In both cases, the diffusion coefficient decreases as the number of bounded neighbours and the degree of order of the aggregates increase. This is attributable to the fact that as the size of the aggregates increases, the number of free particles decreases. To represent the decrease, an exponential fit was done in both cases; in Fig. 9a and b, the solid line corresponds to this fit.

In summary, we have studied the particle kinetics and aggregation processes in a two-dimensional granular system at constant effective temperature. The essential founds of this experimental analysis are the following: first, we have observed that the diffusion coefficient decreases in time due to the formation and growth of clusters at a constant effective temperature. A second one is that the sixth bond-orientational order parameter and the number of bounded neighbours, when considering more than two, are appropriate descriptors for evolution of the degree order of the aggregates. Third, we have observed three regimes of temperature: low, medium, and high. At high temperature the formation of aggregates is hardly observed. At medium and low temperatures, we found the formation of several aggregates. Particularly, at medium temperature the order of the aggregates is higher, the aggregates are crystalline and numerous. In Supplementary Movies 1–3, one observes the temporal evolution of the aggregates at low ( first 30 s), medium (at 300 s), and high temperature (at 300 s), respectively.

The classical theory of nucleation assumes that once the nucleus is formed, the ulterior aggregation process occurs practically as an epitaxial growth, propagating the nucleus crystalline structure. From this analysis, we may conclude that, after the energetic balance that allows the formation of small disordered aggregates, these structures evolve, becoming more compact and ordered. Thus, our observations indicate that there is a threshold condition in which the volumetric potential becomes predominant over the surface potential, propitiating from that point and beyond the formation of disordered aggregates. Then, a second threshold is needed for the flyover to achieve order of these aggregates. In this sense, the growth of the aggregates observed in our experiments is consistent with a two-step nucleation theory^43,44^.

## Methods

To characterise the different structural characteristics’ phases, the radial distribution function (RDF) is commonly used. This quantity is useful when the measured characteristic phase is sufficiently large to capture a robust average by the RDF. The domain of the RDF must be spanned by at least some tens of the aggregate size. When the sample contains small parts of a different phase, their characteristics are not clearly shown in the RDF. To deal with small aggregates, some other quantities have been developed. That is the case with the sixth bond-orientational order parameter, which is used when aggregates tend to be ordered in the hexagonal close-packed arrangement. Several authors have used this parameter to quantify the two-dimensional crystallisation process^45–47^. It also allows us to identify phase transitions—for instance, from a liquid to a crystalline lattice^48,49^.

**Figure 10:**
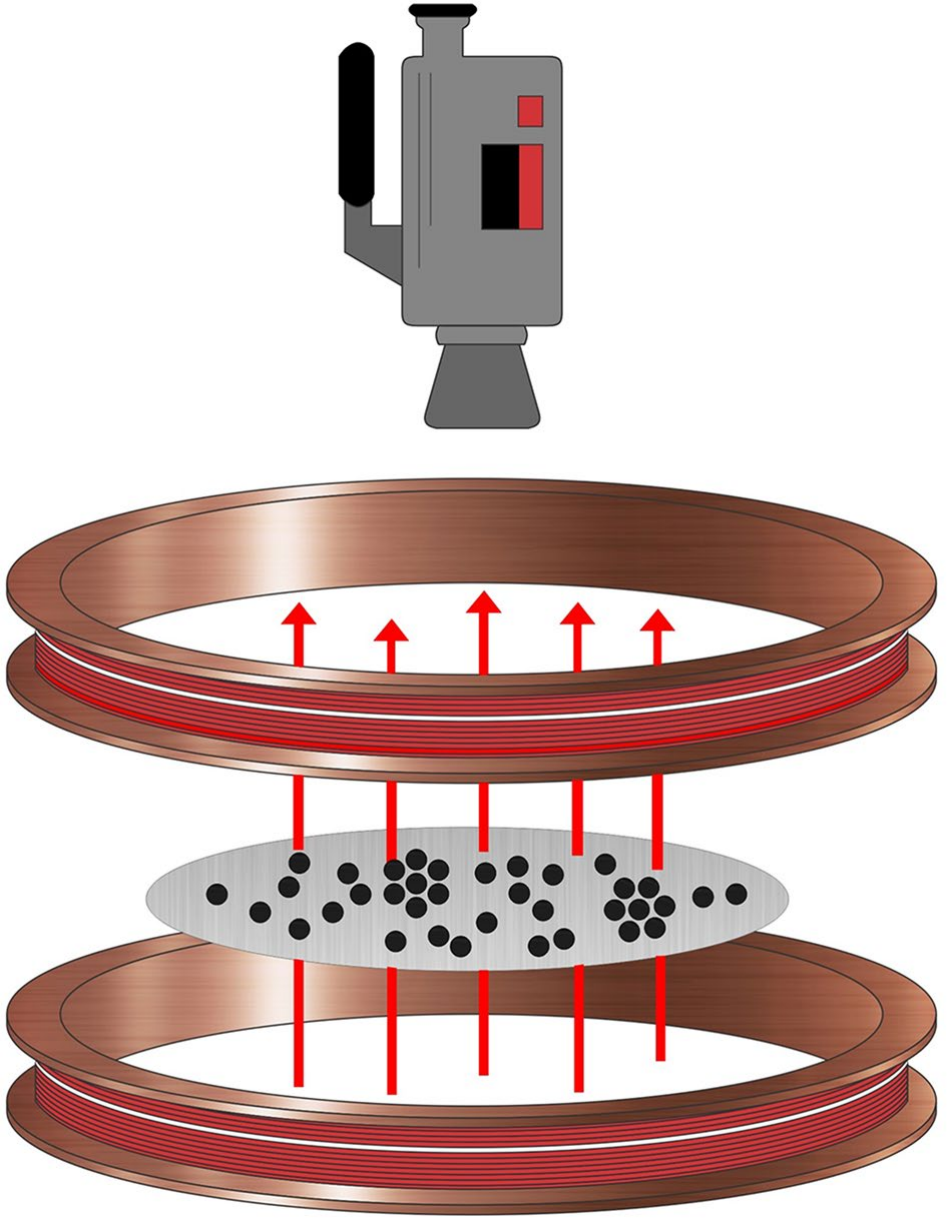
Schematic representation of the experimental setup for a nonvibrating granular system.

To prepare our system, we used 1050 spherical particles that settled on the horizontal glass surface bounded by a circular wall of 70 mm in diameter. The particle concentration is the area fraction occupied by the particles $\phi _{2D}$=0.19. The particles are steel balls of 1 mm in diameter, ANSI 420 grade 1000 by Gimex S.A. The glass plane is allocated in the centre of a pair of Helmholtz coils, which produce a vertical magnetic field. The coils are fed by a Kepco BOP 36-6 M power amplifier. This system of magnetic spheres is subjected to a sinusoidal magnetic field $B=B_{0}\sin {(2\pi ft)}$, whose amplitude ($B_{0}$) was kept constant during each experiment and takes values from 22 to 50 G. We carried out three sets of experiments: series 1, series 2 and series 3. The frequency (*f*) was fixed at 9.24 Hz. The applied magnetic field plays an effective temperature role. Henceforth, we refer to the amplitude of the magnetic field as the effective temperature^24,40^. Figure 10 shows the experimental setup.

The dynamics of the system are explained as follows. Each particle has a magnetic moment. As the magnetic field changes its direction (up and down), a particle’s magnetic moment tends to change its direction following the magnetic field’s direction, and minimising its potential energy. When the particle’s magnetic moment points opposite to the field direction, it is in a very unstable state. Thus, it rotates in a random direction to align with the magnetic field. In following the magnetic field direction, the particle rotates and rolls, experiencing repulsive contact forces due to the other particles that tend to separate them.

**Figure 11:**
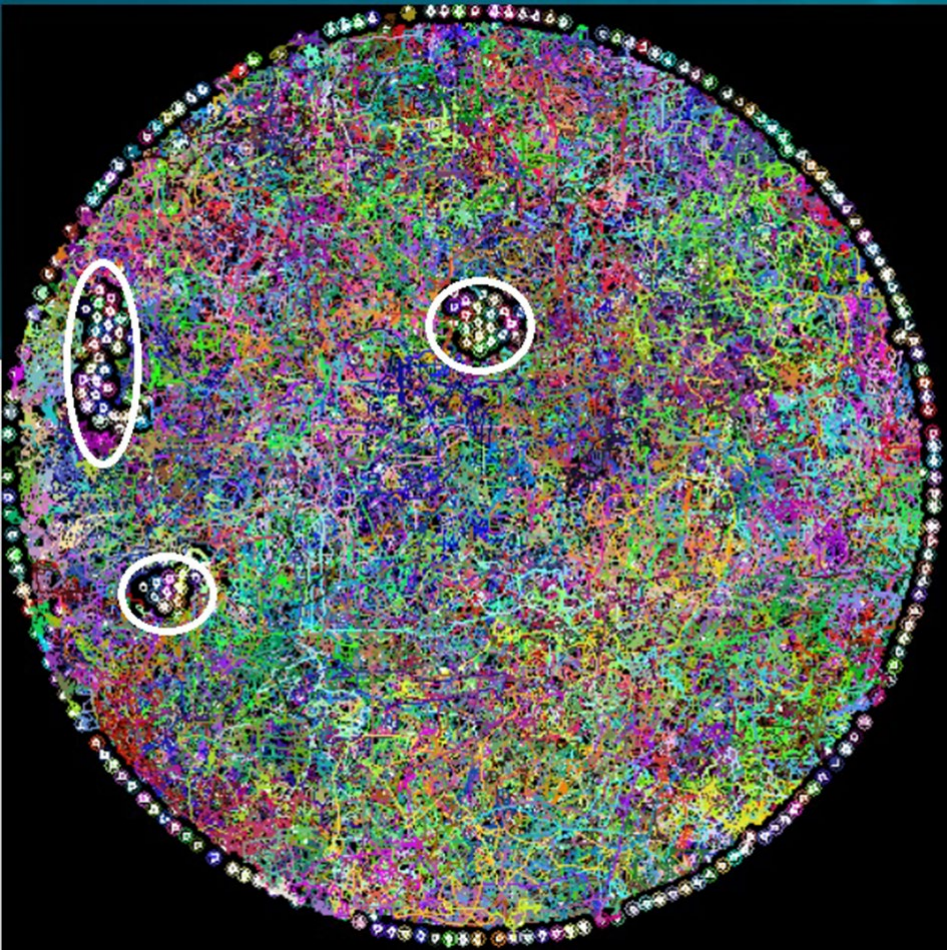
Coloured lines are for particle’s trajectories at 44.09 G. Aggregates are enclosed by white ellipses.

A HandyCam digital camera was used to record the dynamics of the system. We obtained 5 mins of the video at a standard resolution of 720,540 pixels at 30 fps in AVI interlaced format. At this capture rate, the particle positions were not always well defined; thus, we used a filter to deinterlace the video frames to obtain well-defined image sequences with a resolution of $\tau =1/60$s. Subsequently, the image sequences underwent a cleaning process to calculate the particles’ trajectories using the program ImageJ and its plugin Mosaic^50,51^. The mean square displacement was calculated from these trajectories. Figure 11 shows the superimposed trajectories of all the particles during a time interval for the case when the magnetic field’s amplitude is 44 G. Those particles on the circular boundary do not collide with other particles, only they slightly move about their positions. Similarly, particles that form stable and ordered aggregates, such as those enclosed in white circles in Fig. 11, do not move either. Only free particles can move over the entire surface. In the case of Fig. 11, the effective temperature is high, so the particles follow long trajectories. The diffusion coefficient, *D*, was obtained by the Einstein fluctuation-dissipation relation in a diffusive two-dimensional system,$<\Delta r^{2}\hbox {(t)}>=4Dt$.

**Figure 12:**
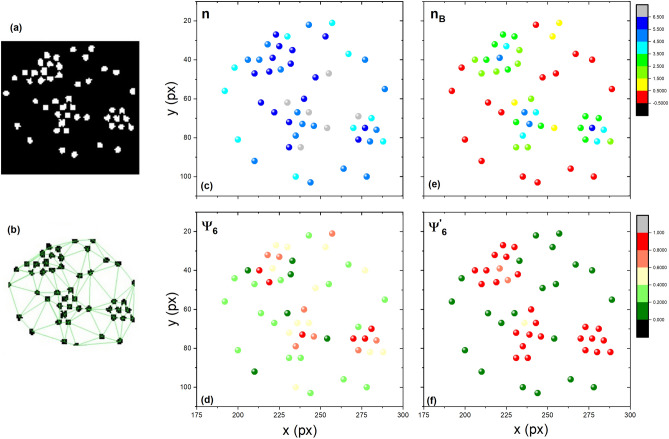
(**a**) Particle configuration after a digital treatment of a photograph. (**b**) Particles are joined by lines forming a Delaunay triangulation. (**c**) Particles are coloured according to the number of neighbours, *n*. (**d**) Particles are coloured according to the sixfold orientational order parameter, $\Psi _{6}$. (**e**) Particles are coloured according to the number of bounded neighbours, $n_B$. (**f**) Particles are coloured according to the sixfold bond orientational order parameter, determined by the use of the bounded neighbours, $\Psi ^{'}_{6}$.

To determine structural parameters, particles are first detected and separated from each other by using ImageJ and some of its plugins—see Fig. 12a. The plugin Delaunay-Voronoi in ImageJ was used to generate the Delaunay triangulation, as shown in Fig. 12b. Lastly, the sixth bond-orientational order parameter is calculated by using the expression:

1 $$\begin{aligned} \Psi _{6}=\frac{1}{n_i}\sum _{j=1}^{n_i}\exp ({\text {i}6\theta _{ij}}), \end{aligned}$$

where $\theta _{ij}$ is the angle between the line formed by the reference particle *i* and neighbour *j* and the x-axis, and $n_i$ is the number of neighbours determined on the basis of the Delaunay triangulation^27^. The value of the average of the sixth bond-orientational order parameter, $<\Psi _{6}>$, tells us whether an aggregate is stable and ordered or if it is in a disordered phase—i.e., if $<\Psi _{6}>$= 1, the aggregates form perfect hexagonal networks, and if $<\Psi _{6}>$= 0, it is a strongly disordered system.

Figure 12c shows the number of neighbours, *n*, for a particle array, and Fig. 12d shows the sixth bond-orientational order parameter $\Psi _{6}$. These parameters did not fully represent the hexagonal compact arrangement. Therefore, the bounded neighbours $n_B$ calculation and the sixfold bond-orientational order parameter from the bounded neighbours $\Psi ^{'}_{6}$ were introduced. These quantities are represented in Fig. 12e and f, respectively. The bounded neighbours were determined considering the nearest neighbours within a distance of one particle diameter. The sixth bond-orientational order parameter $\Psi ^{'}_{6}$ is calculated considering only the bounded neighbours.

## Supplementary Information

Supplementary Movie 1.

Supplementary Movie 2.

Supplementary Movie 3.
